# Fatal Post-Influenza Streptococcal Toxic Shock Syndrome Caused by M1_UK_ *Streptococcus pyogenes* in South Korea: A Case Report

**DOI:** 10.3390/microorganisms14102373

**Published:** 2026-10-16

**Authors:** Hae Seong Jeong, Yong Jun Kwon, Mihee Kim, Joon-Young Yoon, Yong Hun Jung, Soo Hyun Kim

**Affiliations:** 1 Department of Infectious Diseases, Chonnam National University Medical School, Gwangju 61469, Republic of Korea; tndmsemd21@naver.com; 2 Department of Laboratory Medicine, Chonnam National University Hospital, Chonnam National University Medical School, Gwangju 61469, Republic of Korea; 3 Department of Hematology-Oncology, Chonnam National University Hwasun Hospital, Chonnam National University Medical School, Hwasun 58128, Republic of Korea; mihi4300@gmail.com; 4 Department of Internal Medicine, Division of Pulmonology, Chonnam National University Hospital, Gwangju 61469, Republic of Korea; owlstar@hanmail.net; 5 Department of Emergency Medicine, Chonnam National University Hospital, Chonnam National University Medical School, Gwangju 61469, Republic of Korea; xnxn77@hanmail.net; 6 Department of Laboratory Medicine, Chonnam National University Hwasun Hospital, Hwasun 58128, Republic of Korea; alpinboy@chonnam.ac.kr; 7 Department of Microbiology and Immunology, Chonnam National University Medical School, Hwasun 58128, Republic of Korea

**Keywords:** *Streptococcus pyogenes*, streptococcal toxic shock syndrome, M1_UK_, influenza, whole-genome sequencing

## Abstract

*Streptococcus pyogenes* (*S. pyogenes*) M1_UK_ is an emergent *emm1* lineage associated with increased production of the superantigen SpeA and has spread internationally. We report fatal post-influenza streptococcal toxic shock syndrome (STSS) caused by M1_UK_ *S. pyogenes* in a 49-year-old man in South Korea. Following a diagnosis of influenza A, the patient developed rapidly progressive shock and respiratory failure and died approximately 22 h after hospital presentation despite antimicrobial therapy and intensive organ support. *S. pyogenes* was isolated from blood cultures. Short-read whole-genome sequencing identified the isolate as *emm1* and demonstrated all 27 M1_UK_ lineage-defining single-nucleotide polymorphisms. The isolate carried *speA*, and no acquired macrolide–lincosamide resistance determinants were detected. Comparative core-genome phylogenetic analysis placed the isolate within the international M1_UK_ lineage, whereas Korean pre-COVID-19 *emm1* comparator genomes belonged to the M1_global_ lineage. This case documents a rapidly fatal post-influenza STSS associated with genomically confirmed M1_UK_ *S. pyogenes*. It highlights the need to consider STSS and adjunctive antitoxin therapy in patients with shock after influenza, even when the presentation is pulmonary, and supports continued genomic surveillance of invasive *S. pyogenes* in Korea.

## 1. Introduction

The M1_UK_ lineage of *Streptococcus pyogenes* (*S. pyogenes*), first described in England in 2019, is an emergent *emm1* variant defined by 27 core-genome single-nucleotide polymorphisms (SNPs) and associated with increased production of the superantigen SpeA [1]. It has expanded across Europe, North America, and Oceania and has been reported in East Asia, including Japan and China [2,3]. Post-pandemic increases in invasive group A streptococcal (iGAS) disease, accompanied by expansion of M1_UK_, have been documented by national surveillance in the United Kingdom and the Netherlands [4,5] and have prompted calls for enhanced molecular surveillance in Korea [6]; yet detailed genomic characterization of invasive M1_UK_ isolates from Korea remains limited.

A pre-COVID-19 Korean analysis found no M1_UK_ among 17 archived *emm1* genomes collected during 1997–2018 [7]. Two M1_UK_ isolates were subsequently identified in Korea through a nationwide surveillance study—an adult brain abscess case in 2020 and a pediatric case of streptococcal toxic shock syndrome with necrotizing fasciitis in 2024—and a separate report identified M1_UK_ by whole-genome sequencing (WGS) in two adolescents with fatal toxic shock syndrome in 2025, one of whom had preceding influenza B [8,9]. This report describes fatal post-influenza streptococcal toxic shock syndrome (STSS) caused by M1_UK_ *S. pyogenes* in a Korean adult and places the invasive isolate within a global genomic context. This case report was prepared in accordance with the CARE guidelines [10]; the completed CARE checklist is provided in Supplementary File S1.

## 2. Case Presentation

On 15 December 2025, a 49-year-old man presented to Chonnam National University Hospital (CNUH), Gwangju, Republic of Korea. Earlier that day, he had been diagnosed with influenza A by PCR at a local hospital after presenting with fever and sputum production and had received a single dose of intravenous peramivir. He had not been vaccinated against influenza for the 2025–2026 season and was not eligible for publicly funded vaccination under the Korean National Immunization Program. Fever and sputum production had begun on 12 December 2025. He subsequently developed hemoptysis and progressive dyspnea. On arrival, he was alert but critically ill, with a heart rate of 120 beats/min, systolic blood pressure of 80 mmHg, and oxygen saturation of 80% on room air. Subtle erythematous change was present on the trunk, without a focal soft-tissue source. His only notable medical history was intermittent arthralgia, mainly of the knees, for which, according to his family, he intermittently took loxoprofen, a nonsteroidal anti-inflammatory drug (NSAID), one tablet three times daily; osteoarthritis had not been formally diagnosed. He had no history of immunosuppressive therapy or recent surgery. He had a 30 pack-year smoking history and drank alcohol socially. No relevant family history was reported. Laboratory testing showed profound leukopenia (white blood cell count, 0.5 × 10^3^/µL; absolute neutrophil count, 0.08 × 10^3^/µL), elevated C-reactive protein (22.6 mg/dL), procalcitonin (40.3 ng/mL), presepsin (3402 pg/mL), and lactate (8.22 mmol/L). The platelet count was 165 × 10^3^/µL, serum ferritin 80 ng/mL, prothrombin time–international normalized ratio (PT-INR) 1.09, fibrinogen 625.7 mg/dL (reference, 180–400 mg/dL), D-dimer 2.97 mg/L (reference, 0–0.55 mg/L), and serum creatinine 1.92 mg/dL. Chest computed tomography showed diffuse bilateral consolidation without pleural effusion. Blood cultures were obtained within 15 min of arrival, and empiric piperacillin–tazobactam plus vancomycin was started within 30 min. Influenza testing was not repeated at CNUH, and no further antiviral therapy was administered after arrival.

**Figure 1:**
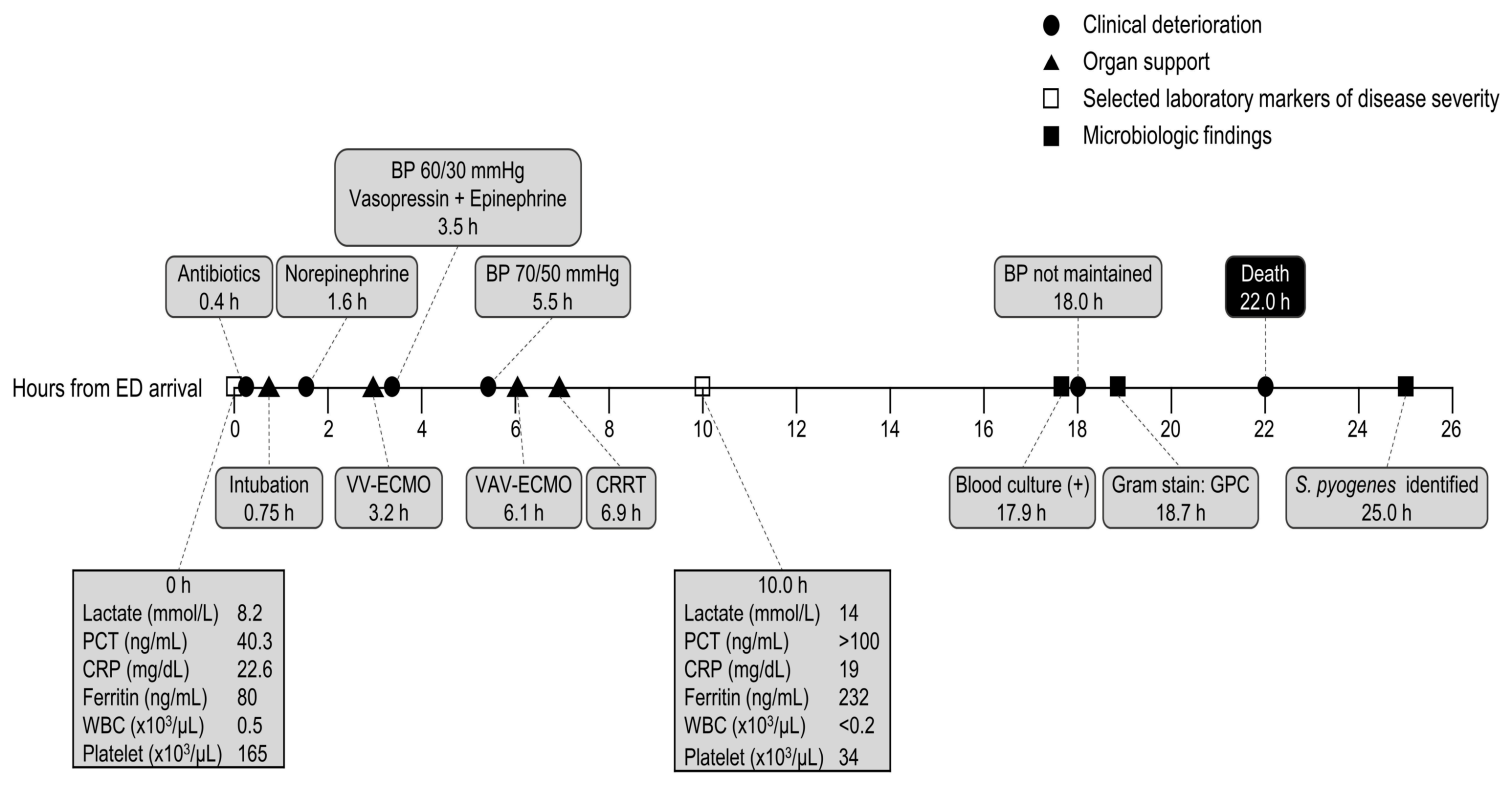
Clinical course and laboratory findings in a fatal case of post-influenza streptococcal toxic shock syndrome caused by M1_UK_ *Streptococcus pyogenes*, Republic of Korea, 2025. The timeline begins at emergency department (ED) arrival on 15 December 2025 and shows rapid clinical deterioration, escalation of organ support, selected laboratory findings, death at 22.0 h and microbiological results through species identification at 25.0 h. Organ support included intubation, venovenous extracorporeal membrane oxygenation (VV-ECMO), conversion to venoarterial–venous extracorporeal membrane oxygenation (VAV-ECMO), and continuous renal replacement therapy (CRRT). Microbiological findings included blood culture positivity, detection of Gram-positive cocci (GPC) on Gram stain, and identification of *S. pyogenes*. Selected laboratory markers measured at ED arrival (0 h) and 10 h included lactate, procalcitonin (PCT), C-reactive protein (CRP), ferritin, white blood cell (WBC) count, and platelet count.

Despite antimicrobial therapy, vasopressors, stress-dose hydrocortisone, intubation, venovenous followed by venoarterial–venous extracorporeal membrane oxygenation, and continuous renal replacement therapy, the patient developed disseminated intravascular coagulation (DIC) and multiorgan failure and died approximately 22 h after arrival, on 16 December 2025 (Figure 1). By 10 h, the platelet count had decreased to 34 × 10^3^/µL despite platelet transfusion and the white blood cell count to <0.2 × 10^3^/µL, whereas ferritin had increased to 232 ng/mL; on follow-up testing, the PT-INR had increased to 2.94 and serum creatinine to 2.38 mg/dL. Fibrinogen and D-dimer were not repeated. Using the admission fibrinogen and D-dimer values, the International Society on Thrombosis and Haemostasis overt DIC score was at least 6 (platelet count, 2; prolonged prothrombin time, 2; D-dimer, 2; fibrinogen, 0) [11]. Packed red blood cells (2 units) and fresh frozen plasma (2 units) were transfused in the emergency department, and platelet concentrates (8 units) were transfused immediately before extracorporeal membrane oxygenation (ECMO) cannulation; anticoagulation during ECMO was maintained with intravenous unfractionated heparin. Granulocyte colony-stimulating factor was administered at approximately midnight on 16 December 2025 for profound neutropenia; granulocyte transfusion was not performed. Blood cultures became positive at 17.9 h, and Gram-positive cocci were detected on Gram stain at 18.7 h; *S. pyogenes* was identified at 25.0 h, approximately 3 h after death. The presentation fulfilled the consensus definition of confirmed STSS [12]: isolation of *S. pyogenes* from blood, hypotension (systolic blood pressure, 80 mmHg), and at least two organ-system criteria, namely renal impairment (serum creatinine, 2.38 mg/dL, requiring continuous renal replacement therapy [CRRT]), coagulopathy (platelet count, 34 × 10^3^/µL, with overt DIC), and acute respiratory distress syndrome (diffuse bilateral consolidation with refractory hypoxemia requiring ECMO). The truncal erythema was subtle and was not recognized as a toxin-mediated rash at presentation. At presentation, the differential diagnosis comprised severe influenza pneumonia with acute respiratory distress syndrome; secondary bacterial pneumonia with septic shock, principally caused by *Staphylococcus aureus* (including methicillin-resistant strains), *Streptococcus pneumoniae*, or *S. pyogenes*; STSS; and diffuse alveolar hemorrhage, given the hemoptysis. Empiric piperacillin–tazobactam plus vancomycin was selected to cover secondary bacterial pneumonia. Adjunctive clindamycin and intravenous immunoglobulin were not administered, as STSS was not the leading diagnostic consideration during the early clinical course; the pulmonary presentation, absence of a soft-tissue focus, and preceding diagnosis of influenza had initially favored severe viral–bacterial pneumonia. A patient perspective could not be obtained because of the rapidly fatal course.

## 3. Microbiological and Genomic Characterization

### 3.1. Microbiological Investigation

The bloodstream isolate was identified as *S. pyogenes* by matrix-assisted laser desorption/ionization time-of-flight mass spectrometry using the MicroIDSys system (ASTA, Suwon, Republic of Korea). Antimicrobial susceptibility testing was performed using the VITEK 2 system (bioMérieux, Marcy-l’Étoile, France), including automated assessment of inducible clindamycin resistance. Results were interpreted according to Clinical and Laboratory Standards Institute M100-Ed35 criteria [13]. The isolate was susceptible to penicillin (minimum inhibitory concentration [MIC], ≤0.06 µg/mL), erythromycin (MIC, ≤0.12 µg/mL), and clindamycin (MIC, ≤0.25 µg/mL). Automated testing for inducible clindamycin resistance using VITEK 2 was negative.

### 3.2. Whole-Genome Sequencing and Bioinformatic Analysis

Whole-genome sequencing was performed using the NovaSeq X platform (Illumina, San Diego, CA, USA). Sequence reads were quality-trimmed and assembled using CLC Genomics Workbench v26.0.1 (QIAGEN, Aarhus, Denmark). In silico *emm* typing was performed using the CDC *emm* sequence database. Sequence reads were mapped to the M1 reference strain MGAS5005 (GenBank accession no. NC_007297.2). Mapping quality was assessed against the MGAS5005 reference genome, and breadth of coverage at ≥10× depth was calculated from the read mapping using SAMtools v1.22.1. Variants were identified using the Basic Variant Detection tool in CLC Genomics Workbench v26.0.1 with a minimum coverage of 10×, a minimum supporting read count of 2, and a minimum variant frequency of 35%. Variant calls were compared with the published set of 27 M1_UK_ lineage-defining SNPs for lineage assignment. Virulence-associated genes were screened in the short-read draft genome assembly using ABRicate v1.0.1 against the Virulence Factor Database (VFDB; 2597 sequences; accessed 6 October 2026), retaining hits with both ≥90% nucleotide identity and ≥90% sequence coverage. Antimicrobial resistance determinants were screened in the assembled genome using AMRFinderPlus v4.2.7 with database version 2026-05-15.1 and the *Streptococcus pyogenes* organism option.

For phylogenetic analysis, 55 publicly available *emm1 S. pyogenes* genomes were selected to provide lineage and geographic context for the study isolate rather than to represent all available *emm1* genomes. The comparator set included accessible Korean pre-COVID-19 *emm1* genomes and representative M1_global_, M1_intermediate_, and M1_UK_ genomes from East Asia and other regions. The recently reported Korean M1_UK_ isolates were not included because the corresponding sequence data were not publicly available. A core-genome SNP alignment was generated using Snippy v4.6.0 with MGAS5005 (NC_007297.2) as the reference, and recombinant regions were removed using Gubbins v3.4.3. ModelFinder implemented in IQ-TREE v2.4.0 selected K3P + ASC as the best-fit substitution model according to the Bayesian information criterion. A maximum-likelihood phylogenetic tree was inferred under this model, with branch support assessed using 1,000 ultrafast bootstrap replicates. The analysis was performed as an unrooted maximum-likelihood phylogeny; MGAS5005 was included as the reference genome. The final tree comprised the study isolate C22690, 55 public comparator genomes from eight countries, and the reference strain MGAS5005. The study isolate and comparator genomes, together with their metadata, are listed in Supplementary Table S1.

### 3.3. Genomic and Phylogenetic Findings

The short-read draft assembly of the bloodstream isolate C22690 comprised 30 contigs, with a total length of 1,839,905 bp and an N50 of 221,350 bp. Reference-based mapping yielded a mean read depth of 1910×, with 99.996% reference genome coverage. C22690 was assigned to emm1, and all 27 M1_UK_ lineage-defining SNPs were detected, supporting assignment to the M1_UK_ lineage (Supplementary Table S2). VFDB screening identified 25 virulence-associated genes, including the superantigen genes *speA*, *speC*, *speG*, *speJ*, and *smeZ*, as well as *speB*, *slo*, and the DNase genes *mf*/*spd*, *mf2*, and *mf3* (Supplementary Table S3). AMRFinderPlus detected no acquired macrolide–lincosamide resistance determinants, consistent with the phenotypic susceptibility to erythromycin and clindamycin.

**Figure 2:**
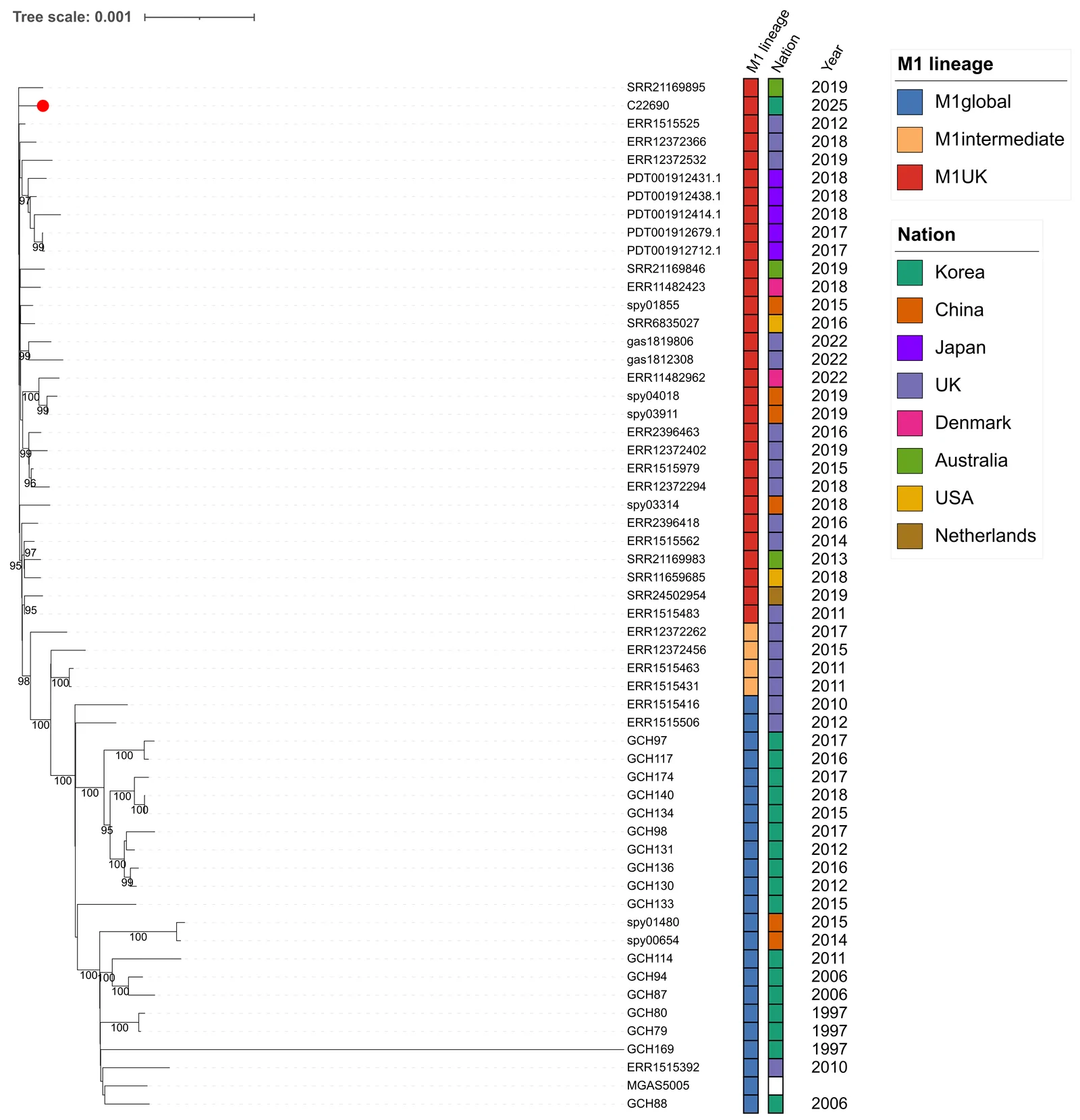
Maximum-likelihood phylogenetic analysis of *emm1 Streptococcus pyogenes*. The phylogeny includes the study isolate C22690, 55 publicly available comparator genomes from eight countries, and the reference strain MGAS5005 (NC_007297.2). A core-genome SNP alignment was generated using Snippy v4.6.0 with MGAS5005 as the reference, and recombinant regions were identified and removed using Gubbins v3.4.3. ModelFinder implemented in IQ-TREE v2.4.0 selected K3P + ASC as the best-fit substitution model according to the Bayesian information criterion. An unrooted maximum-likelihood phylogenetic tree was inferred under this model with 1000 ultrafast bootstrap replicates; support values ≥ 95% are displayed. The study isolate C22690 is indicated by a red circle. M1 lineage, country, and year of isolation are shown alongside the tree. The M1_intermediate_ category comprises the M1_13SNP_ and M1_23SNP_ intermediate lineages; specific intermediate-lineage assignments are provided in Supplementary Table S1. The scale bar indicates substitutions per site.

In the recombination-filtered core-genome SNP phylogeny, C22690 was positioned within the M1_UK_ lineage together with isolates from multiple geographic regions, with 98% ultrafast bootstrap support for the C22690-containing M1_UK_ clade (Figure 2). In contrast, the Korean *emm1* genomes collected during the pre-COVID-19 period were assigned to the M1_global_ lineage. Four comparator genomes represented intermediate M1 lineages: BHS0076 and gas83625 were classified as M1_13SNP_, whereas BHS0108 and gas392701 were classified as M1_23SNP_. For visualization, both intermediate lineages were grouped as M1_intermediate_ in Figure 2, while their specific lineage assignments are retained in Supplementary Table S1. These findings independently supported the M1_UK_ lineage assignment of C22690 and placed the Korean invasive isolate within the broader international M1_UK_ genomic context.

## 4. Discussion

This report describes fulminant post-influenza STSS caused by an M1_UK_ *S. pyogenes* bloodstream isolate in an adult in the Republic of Korea. The patient progressed from emergency department presentation to death within 22 h despite antimicrobial therapy and intensive organ support. Genomic analysis demonstrated all 27 M1_UK_ lineage-defining SNPs and placed the isolate within the international M1_UK_ lineage on core-genome phylogenetic analysis. Earlier Korean M1_UK_ reports comprised an adult brain abscess in 2020 and a pediatric case of STSS with necrotizing fasciitis in 2024, both identified in a multicenter study [8], and two fatal cases of toxic shock syndrome in adolescents in 2025, one of which followed influenza B [9]. To our knowledge, the present case is the first genomically characterized fatal M1_UK_ STSS in an adult in Korea. It adds three elements to these reports: an influenza A-associated pulmonary presentation in an adult, a detailed timeline relating microbiological results to clinical deterioration, and publicly available sequence data placed in an international phylogenetic context. Sequence data from the earlier Korean cases were not publicly available and therefore could not be included in our phylogeny.

Antecedent influenza may increase susceptibility to invasive *S. pyogenes* infection through disruption of respiratory epithelial barriers and altered host–pathogen interactions [14,15]. In this patient, the initial pulmonary presentation, bilateral consolidation, and preceding diagnosis of influenza A favored severe viral–bacterial pneumonia, while subtle truncal erythema, profound leukopenia, refractory shock, and rapid multiorgan failure subsequently supported STSS. Intermittent NSAID use may have attenuated fever and local inflammatory signs and thereby obscured the severity of the evolving infection; in community-acquired pneumonia, NSAID exposure before admission has been associated with a delayed and atypical presentation and more frequent pleuropulmonary complications [16]. The antiplatelet effect of NSAIDs may also have contributed to hemoptysis. Because hemoptysis began before admission, when the platelet count and PT-INR were within reference ranges, thrombocytopenia and overt coagulopathy are unlikely to account for its onset; pulmonary infection may also have contributed, although early activation of coagulation, suggested by the elevated admission D-dimer concentration, cannot be excluded. The individual contributions of antecedent influenza and NSAID exposure to the fulminant course therefore cannot be determined from a single case. The temporal laboratory pattern was also notable: between arrival and 10 h, lactate increased from 8.2 to 14 mmol/L, procalcitonin from 40.3 to >100 ng/mL, and ferritin from 80 to 232 ng/mL, whereas C-reactive protein did not increase (22.6 to 19 mg/dL); over the same interval, the platelet count fell from 165 to 34 × 10^3^/µL despite transfusion of platelet concentrates, consistent with evolving consumptive coagulopathy. Given the absence of baseline measurements and the short observation period, however, this discordance should not be interpreted as evidence of a specific toxin-mediated biomarker pattern. The admission presepsin concentration (3402 pg/mL) was markedly elevated and consistent with severe bacterial sepsis; however, presepsin is cleared by the kidneys and increases in acute kidney injury [17], so its prognostic weight in this patient, who required CRRT within 7 h of arrival, is uncertain.

In retrospect, several features present at arrival, namely shock (systolic blood pressure, 80 mmHg), hypoxemia (oxygen saturation, 80% on room air), profound leukopenia (white blood cell count, 0.5 × 10^3^/µL), a lactate concentration of 8.22 mmol/L, and subtle truncal erythema, were warning signs of toxin-mediated disease. Among the bacteria that cause severe post-influenza pneumonia, *S. aureus* and *S. pyogenes* both produce superantigens and other exotoxins. Therefore, when a patient with recent viral infection presents with shock and features suggesting toxin-mediated disease, empiric antitoxin therapy should be considered on clinical grounds, even when the presentation is pulmonary, no soft-tissue focus is evident, and the causative organism has not yet been identified. Protein synthesis inhibitors suppress exotoxin production by both organisms; in a large multi-center cohort, adjunctive clindamycin was associated with lower mortality among β-lactam-treated patients with invasive group A streptococcal infection [18], and polyspecific intravenous immunoglobulin may provide additional benefit in clindamycin-treated STSS [19]. C22690 was susceptible to clindamycin (MIC, ≤0.25 µg/mL), with negative automated testing for inducible resistance, so this approach would have been microbiologically active. By contrast, the laboratory timeline offered little opportunity: blood cultures became positive at 17.9 h and Gram-positive cocci were reported at 18.7 h, whereas species identification was available only at 25.0 h, after death. Rapid identification by MALDI-TOF mass spectrometry from short-incubation subcultures of positive blood-culture broth can shorten this interval [20], but blood pressure could no longer be maintained from 18.0 h. The main opportunity therefore lay in clinical recognition of possible toxin-mediated disease at presentation, although whether earlier antitoxin therapy would have altered the outcome of this fulminant illness cannot be determined.

The genomic findings should be interpreted in the context of the evolving epidemiology of *emm1 S. pyogenes* in Korea. Previously available Korean pre-COVID-19 *emm1* genomes were assigned to the M1_global_ lineage, whereas M1_UK_ isolates have subsequently been reported in Korean invasive infections [7,8,9]. In Korea, invasive GAS infection has not been a nationally notifiable disease, and epidemiological data derive mainly from hospital-based studies. A multicenter study of 454 patients from 23 university hospitals during 2015–2024 documented a marked decline in invasive GAS infection during the COVID-19 pandemic, followed by a rebound to pre-pandemic levels in children and a smaller increase in adults; approximately one-fifth of patients developed STSS, of whom more than half died [8]. Molecular characterization in that study was limited to available isolates. In the present analysis, C22690 carried the complete set of 27 M1_UK_-defining SNPs and was positioned within the international M1_UK_ lineage. However, the phylogenetic placement of a single isolate cannot establish the prevalence, transmission dynamics, or population-level circulation of M1_UK_ in Korea.

Prevention also deserves emphasis. Respiratory viral infections were estimated to account for approximately one-quarter of iGAS pneumonia and sepsis cases in adults in the Netherlands during the 2022–2023 upsurge [21], and influenza activity is temporally associated with iGAS at the population level [15]. This patient had not been vaccinated against influenza; at 49 years of age, he was not eligible for publicly funded vaccination under the Korean National Immunization Program, which in the 2025–2026 season covered children aged 6 months to 13 years, pregnant women, and adults aged ≥ 65 years. Prevention of antecedent respiratory viral infections, including by influenza vaccination, may therefore contribute to reducing the risk of post-viral iGAS.

This report has several limitations. It describes a single patient and a single bacterial isolate, and the comparator dataset was intentionally selected to provide lineage and geographic context rather than to constitute a population-representative collection. Public genome availability and differences in sampling intensity among countries may therefore influence the apparent phylogenetic context. In addition, functional assays of SpeA production were not performed. Influenza A subtype information was unavailable, and the diagnosis made at the local hospital was not independently confirmed at our hospital. Clinical information was obtained retrospectively from the medical records, and laboratory measurements were available at only two time points before death. Nevertheless, the combination of a rapidly fatal post-influenza clinical course, genomic confirmation of the M1_UK_ lineage, and phylogenetic comparison with Korean and international *emm1* genomes illustrates the value of genomic characterization of invasive *S. pyogenes* and supports continued genomic surveillance to define the distribution of M1_UK_ in Korea.

## Data Availability

The raw sequencing reads for the study isolate C22690 are available in the NCBI Sequence Read Archive under BioProject PRJNA1447141, BioSample SAMN56895991, and SRA accession SRR37900183. Publicly available comparator genomes and their accession numbers are listed in Supplementary Table S1.

## Supplementary Materials

The following supporting information can be downloaded at: https://www.mdpi.com/article/10.3390/microorganisms14102373/s1, Table S1. Genome accession numbers and metadata for the study isolate and publicly available *emm1 Streptococcus pyogenes* comparator genomes used in the phylogenetic analysis; Table S2. Read-level support for the 27 M1_UK_ lineage-defining SNPs in *Streptococcus pyogenes* isolate C22690; Table S3. Virulence-associated genes identified in the short-read draft genome assembly of *Streptococcus pyogenes* C22690 by ABRicate/VFDB screening; File S1. CARE checklist.

## Abbreviations

The following abbreviations are used in this manuscript:

STSS	Streptococcal toxic shock syndrome
DIC	Disseminated intravascular coagulation
WGS	Whole-genome sequencing
SNP	Single-nucleotide polymorphism
VV-ECMO	Venovenous extracorporeal membrane oxygenation
VAV-ECMO	Venoarterial–venous extracorporeal membrane oxygenation
CRRT	Continuous renal replacement therapy
