# Supplementary material for: Fatal Post-Influenza Streptococcal Toxic Shock Syndrome Caused by M1UK Streptococcus pyogenes in South Korea: A Case Report
Source: Microorganisms. 2026 Oct 16;14(10):2373. doi: 10.3390/microorganisms14102373 (PMC13707781; doi:10.3390/microorganisms14102373)
Supplement: Supplementary file 1 [file microorganisms-14-02373-s001.zip › CARE_checklist_final.pdf]

Supplementary File S1. CARE checklist

Fatal Post-Influenza Streptococcal Toxic Shock Syndrome Caused by $M1_{UK}$ *Streptococcus pyogenes* in South Korea: A Case Report. Checklist items follow Gagnier et al., J Clin Epidemiol 2014;67:46–51 (CARE 2013).

Item	Topic	Checklist item description	Location in manuscript
1	Title	The words “case report” appear in the title, with the area of focus	Title (lines 2–4)
2	Key words	2–5 key words identifying diagnoses or interventions	Keywords (lines 38–39)
3a	Abstract	Introduction: what is unique about this case and what it adds to the literature	Abstract, lines 22–25
3b	Abstract	Main symptoms and important clinical findings	Abstract, lines 25–28
3c	Abstract	Main diagnoses, therapeutic interventions, and outcomes	Abstract, lines 25–33
3d	Abstract	Conclusion: main take-away lessons	Abstract, lines 33–37
4	Introduction	Brief background summary of the case, referencing the relevant medical literature	Section 1, lines 42–60
5a	Patient information	De-identified demographic and other patient-specific information	Section 2, lines 63–64
5b	Patient information	Main concerns and symptoms	Section 2, lines 63–70
5c	Patient information	Medical, family, and psychosocial history, including relevant comorbidities	Section 2, lines 73–78 (intermittent arthralgia; 30 pack-year smoking; social alcohol use; no relevant family history)
5d	Patient information	Relevant past interventions and their outcomes	Section 2, lines 64–66 and 73–76 (intravenous peramivir; intermittent loxoprofen; no immunosuppressive therapy or recent surgery)
6	Clinical findings	Relevant physical examination findings	Section 2, lines 70–73
7	Timeline	Relevant data from the episode of care organized as a timeline	Section 2, lines 63–69 and 88–92 (calendar dates); Figure 1, legend lines 126–136 (hourly timeline from ED arrival)
8a	Diagnostic assessment	Diagnostic methods (physical examination, laboratory testing, imaging, surveys)	Section 2, lines 78–86 and 92–98 (including PT-INR, fibrinogen, D-dimer, creatinine); Sections 3.1–3.2, lines 140–182
8b	Diagnostic assessment	Diagnostic challenges	Section 2, lines 105–122; Discussion, lines 236–288
8c	Diagnostic assessment	Diagnostic reasoning, including other diagnoses considered	Section 2, lines 105–121 (STSS criteria; structured differential diagnosis)
8d	Diagnostic assessment	Prognostic characteristics, where applicable	Discussion, lines 251–262 (lactate, procalcitonin, presepsin, platelet trajectory)
9a	Therapeutic intervention	Types of intervention (pharmacologic, surgical, preventive, self-care)	Section 2, lines 84–87, 88–90, and 98–103; Figure 1
9b	Therapeutic intervention	Administration of intervention (dosage, strength, duration)	Section 2, lines 64–66, 73–75, and 98–103; Figure 1 (available administration and timing details)
9c	Therapeutic intervention	Changes in intervention, with rationale	Section 2, lines 88–90 (escalation of organ support; ECMO conversion); Figure 1
10a	Follow-up and	Clinician- and patient-assessed	Section 2, lines 91–92 (death at 22 h)

	outcomes	outcomes	
10b	Follow-up and outcomes	Important follow-up diagnostic and other test results	Section 2, lines 104–105; Sections 3.1–3.3, lines 140–204
10c	Follow-up and outcomes	Intervention adherence and tolerability	Not assessed because of the rapidly fatal course
10d	Follow-up and outcomes	Adverse and unanticipated events	Not specifically documented in the available medical records
11a	Discussion	Strengths and limitations in the approach to this case	Discussion, lines 221–235, 263–288, and 315–327
11b	Discussion	Discussion of the relevant medical literature	Discussion, lines 221–314
11c	Discussion	Rationale for conclusions, including assessment of possible causes	Discussion, lines 236–305
11d	Discussion	Primary take-away lessons	Discussion, lines 263–288 and 306–314; Abstract, lines 33–37
12	Patient perspective	The patient should share their perspective when possible	Not obtainable because of the rapidly fatal course (Section 2, lines 121–122)
13	Informed consent	Did the patient give informed consent?	Informed Consent Statement, lines 345–348 (IRB waiver of consent, including consent for publication of this de-identified case; IRB No. CNUH-EXP-2026-085)
