# Supplementary material for: Fatal Post-Influenza Streptococcal Toxic Shock Syndrome Caused by M1UK Streptococcus pyogenes in South Korea: A Case Report
Source: Microorganisms. 2026 Oct 16;14(10):2373. doi: 10.3390/microorganisms14102373 (PMC13707781; doi:10.3390/microorganisms14102373)
Supplement: Supplementary file 1 [file microorganisms-14-02373-s001.zip › Supplementary_Tables_S1-S3_261012.pdf]

Supplementary Table S1. Genome accession numbers and metadata for the study isolate and publicly available *emm1 Streptococcus pyogenes* comparator genomes used in the phylogenetic analysis

Isolate ID*	Accession no.	Lineage	Nation	Year
C22690	SRR37900183	$M1_{UK}$	Korea	2025
spy01855	JBIZEJ000000000	$M1_{UK}$	China	2015
spy03314	JBIZHF000000000	$M1_{UK}$	China	2018
spy03911	JBIZIE000000000	$M1_{UK}$	China	2019
spy04018	JBIZIP000000000	$M1_{UK}$	China	2019
spy00654	JBIZBI000000000	$M1_{global}$	China	2014
spy01480	JBIZEC000000000	$M1_{global}$	China	2015
GCH79	WSTD00000000	$M1_{global}$	Korea	1997
GCH80	WSTE00000000	$M1_{global}$	Korea	1997
GCH87	WWFL00000000	$M1_{global}$	Korea	2006
GCH88	WWFM00000000	$M1_{global}$	Korea	2006
GCH94	JAAAMZ000000000	$M1_{global}$	Korea	2006
GCH97	JAAANC000000000	$M1_{global}$	Korea	2017
GCH98	JAAAND000000000	$M1_{global}$	Korea	2017
GCH114	JAAANS000000000	$M1_{global}$	Korea	2011
GCH117	JAAANV000000000	$M1_{global}$	Korea	2016
GCH130	JABUOM000000000	$M1_{global}$	Korea	2012
GCH131	JABUON000000000	$M1_{global}$	Korea	2012
GCH133	JABUOP000000000	$M1_{global}$	Korea	2015

GCH134	JABUOQ000000000	$M1_{global}$	Korea	2015
GCH136	JABUJC000000000	$M1_{global}$	Korea	2016
GCH140	JABUJG000000000	$M1_{global}$	Korea	2018
GCH169	JABUAR000000000	$M1_{global}$	Korea	1997
GCH174	JABUAM000000000	$M1_{global}$	Korea	2017
BHS0037	ERR1515392	$M1_{global}$	UK	2010
BHS0061	ERR1515416	$M1_{global}$	UK	2010
BHS0151	ERR1515506	$M1_{global}$	UK	2012
BHS0076	ERR1515431	$M1_{13SNP}$	UK	2011
BHS0108	ERR1515463	$M1_{23SNP}$	UK	2011
BHS0128	ERR1515483	$M1_{UK}$	UK	2011
BHS0170	ERR1515525	$M1_{UK}$	UK	2012
BHS0207	ERR1515562	$M1_{UK}$	UK	2014
BHS0631	ERR1515979	$M1_{UK}$	UK	2015
BHS0749	ERR2396418	$M1_{UK}$	UK	2016
BHS0924	ERR2396463	$M1_{UK}$	UK	2016
M13006969	SRR21169983	$M1_{UK}$	Australia	2013
AUSMDU00062276	SRR21169895	$M1_{UK}$	Australia	2019
AUSMDU00034668	SRR21169846	$M1_{UK}$	Australia	2019
DK_iGAS_2018_0538	ERR11482423	$M1_{UK}$	Denmark	2018
DK_iGAS_2022_0710	ERR11482962	$M1_{UK}$	Denmark	2022
20163759	SRR6835027	$M1_{UK}$	USA	2016
gas1812308	ERR12371640	$M1_{UK}$	UK	2022

gas1819806	ERR12371758	$M1_{UK}$	UK	2022
gas526216	ERR12372294	$M1_{UK}$	UK	2018
gas646170	ERR12372366	$M1_{UK}$	UK	2018
gas705276v2	ERR12372402	$M1_{UK}$	UK	2019
gas956235v2	ERR12372532	$M1_{UK}$	UK	2019
gas83625	ERR12372456	$M1_{13SNP}$	UK	2015
gas392701	ERR12372262	$M1_{23SNP}$	UK	2017
20191426	SRR11659685	$M1_{UK}$	USA	2018
77000009	SRR24502954	$M1_{UK}$	Netherlands	2019
PDT001912712.1	DAUBFP000000000	$M1_{UK}$	Japan	2017
PDT001912679.1	DAUBGX000000000	$M1_{UK}$	Japan	2017
PDT001912438.1	DAUBJX000000000	$M1_{UK}$	Japan	2018
PDT001912431.1	DAUBKD000000000	$M1_{UK}$	Japan	2018
PDT001912414.1	DAUBKU000000000	$M1_{UK}$	Japan	2018

*C22690 was included as the study isolate. Comparison genomes were selected from publicly available *emm1 Streptococcus pyogenes* genomes to provide lineage and geographic context for the study isolate rather than to represent all available *emm1* genomes. The set included accessible Korean pre-COVID-19 *emm1* genomes and representative $M1_{global}$, $M1_{intermediate}$, and $M1_{UK}$ genomes from East Asia and other regions. Recently reported Korean $M1_{UK}$ isolates [8,9] were not included because their corresponding sequence data were not publicly available. Read-level support for the 27 $M1_{UK}$ lineage-defining SNPs in C22690 is provided in Supplementary Table S2.

Supplementary Table S2. Read-level support for the 27 $M1_{UK}$ lineage-defining SNPs in *Streptococcus pyogenes* isolate C22690

MGAS5005 position*	Reference allele	C22690 allele	Supporting reads	Read depth (×)	Variant allele frequency (%)
115646	C	T	1,927	1,929	99.90
116162	A	C	1,839	1,913	96.13
116163	C	A	1,839	1,913	96.13
250832	T	C	1,748	1,748	100.00
513254	G	T	1,886	1,889	99.84
528360	A	T	1,868	1,871	99.84
563631	G	A	1,877	1,879	99.89
613633	T	C	1,619	1,622	99.82
626494	G	A	1,792	1,794	99.89
661707	G	A	1,751	1,757	99.66
730823	C	T	1,775	1,778	99.83
784467	T	C	1,833	1,834	99.95
819098	G	A	1,627	1,630	99.82
923079	G	A	1,696	1,696	100.00
942633	G	T	1,637	1,637	100.00
983438	G	C	1,725	1,727	99.88
1082253	C	T	1,752	1,755	99.83
1238124	G	A	1,724	1,725	99.94
1238673	G	A	1,908	1,909	99.95

1251193	G	A	1,824	1,826	99.89
1373176	C	A	1,809	1,812	99.83
1407497	C	T	1,940	1,942	99.90
1446116	C	T	1,770	1,773	99.83
1535209	A	G	1,935	1,936	99.95
1702540	C	T	1,775	1,775	100.00
1734749	G	A	1,732	1,736	99.77
1828734	G	A	1,775	1,776	99.94

[*]Positions are relative to the *S. pyogenes* MGAS5005 reference genome (NC_007297.2). Supporting read counts, read depths, and variant allele frequencies were obtained from reference-based read mapping and Basic Variant Detection in CLC Genomics Workbench v26.0.1. Positions 116162 and 116163 were called by CLC as a single multinucleotide variant (AC>CA) and are presented separately according to the published 27-SNP M1UK definition. All 27 lineage-defining positions carried the M1UK-associated allele.

Supplementary Table S3. Virulence-associated genes identified in the short-read draft genome assembly of *Streptococcus pyogenes* C22690 by ABRicate/VFDB screening

Gene*	VFDB product	VFDB accession	Coverage (%)	Identity (%)
speJ	Streptococcal exotoxin J precursor	NP_268735	100.00	100.00
ska	Streptokinase A precursor	NP_269944	100.00	100.00
smeZ	Streptococcal mitogenic exotoxin Z	NP_269959	100.00	100.00
lmb	Laminin-binding surface protein	NP_688239	100.00	98.37
scpA	C5a peptidase precursor	NP_269970	98.56	98.56
emm	M protein type 1	NP_269973	100.00	99.79
speB	Pyrogenic exotoxin B	NP_269985	100.00	100.00
mf/spd	Deoxyribonuclease	NP_269989	100.00	100.00
hasA	Hyaluronate synthase	NP_270107	100.00	99.84
hasB	UDP-glucose 6-dehydrogenase	NP_270108	100.00	100.00
hasC	UDP-glucose pyrophosphorylase	NP_270109	100.00	100.00
speG	Streptococcal exotoxin G precursor	NP_268582	100.00	100.00
cpa	Fibronectin-binding protein GAS pilus tip adhesin	YP_281471	100.00	100.00
lepA	Signal peptidase I (pilus locus)	YP_281472	100.00	100.00
fctA	Major pilin FctA	YP_281473	100.00	100.00

srtC1	Sortase (pilus locus)	YP_281474	100.00	100.00
fctB	Minor pilin FctB	YP_281475	100.00	100.00
slo	Streptolysin O precursor	NP_268546	100.00	98.78
grab	Protein GRAB	NP_269464	100.00	100.00
mf3	Deoxyribonuclease	NP_269520	100.00	100.00
speC	Streptococcal exotoxin C precursor, phage associated	NP_268943	100.00	100.00
mf2	Deoxyribonuclease, phage associated	NP_268944	100.00	100.00
ideS/mac	Immunoglobulin G-degrading enzyme	NP_269065	100.00	100.00
fbp54	Fibronectin-binding protein Fbp54	NP_269190	100.00	100.00
speA	Streptococcal exotoxin A precursor, phage associated	NP_665105	100.00	99.73

*Virulence-associated genes were screened using ABRicate v1.0.1 against the Virulence Factor Database (VFDB; 2,597 sequences; accessed 6 October 2026). Hits meeting both ≥90% nucleotide identity and ≥90% sequence coverage were retained.
